# F27. LATENT PROFILES OF DEVELOPMENTAL SCHIZOTYPY IN THE GENERAL POPULATION: ASSOCIATIONS WITH CHILDHOOD TRAUMA AND FAMILIAL MENTAL ILLNESS

**DOI:** 10.1093/schbul/sby017.558

**Published:** 2018-04-01

**Authors:** Melissa Green, Richard J Linscott, Kristin R Laurens, Stacy Tzoumakis, Kimberlie Dean, Johanna C Badcock, Vaughan J Carr

**Affiliations:** 1 University of New South Wales Prince of Wales Hospital; 2 University of Otago; 3 Australian Catholic University; 4 University of New South Wales; 5 University of Western Australia

## Abstract

**Background:**

Latent liability for schizophrenia (schizotypy) is expressed in various combinations of cognitive, psychological, and behavioural characteristics evident in the general population. Historical models propose that distinct classes of individuals expressing different forms of schizotypy may represent manifestations of differential levels of genetic and environmental risk for schizophrenia (or related illness). However, there has been little investigation of developmental models of schizotypy in childhood. Here, we sought to delineate latent profiles of schizotypy among children aged 11–12 years, and to examine associations between emerging schizotypal profiles and parental history of mental illness (as a proxy for genetic risk), early life trauma, and childhood contact with health services for mental illness up to age 13 years.

**Methods:**

Latent profiles of schizotypy were distinguished among 22,137 children (mean age=11.9 years) for whom intergenerational records of health service contact for mental illness and child protection reports were linked to the Middle Childhood Survey (MCS) within the NSW Child Development Study.1 Selected MCS items were used to index schizotypy across six domains (Unusual Experiences, Cognitive Disorganisation, Impulsive Non-conformity, Introversion, Dysphoria and Self-Other disturbance). Using Latent Profile Analyses (LPA), four groups emerged according to patterns of expression across these domains; membership of three putative schizotypy groups were examined in relation to the likelihood of being exposed to childhood maltreatment and parental mental illness, and the child’s own mental illness up to age 13 years, relative to the no risk group.

**Results:**

Four classes emerged from the LPA: (1) ‘schizotypy’ (n=1323; 6%); (2) ‘dysphoric pseudo-schizotypy’ (n=4261, 19%); (3) ‘introverted pseudo-schizotypy’ (n=4473; 20%) and; (4) ‘no psychopathology’ (no-risk, n=12,080; 55%). Children in the schizotypy group had the greatest odds of being the subject of a child protection report (OR=2.9, 95% CI 2.6–3.3) and in contact with health services for mental illness by age 13 years (OR=2.7, 95% CI 2.2–3.3), relative to the no-risk group. The odds of child protection reports and childhood mental disorders were smaller, yet significantly increased, among dysphoric pseudo-schizotypy (ORs=1.9 and 1.8, respectively) and introverted pseudo-schizotypy (ORs=1.7 and 1.4, respectively), relative to the no-risk group. Parental mental illness exposure was greatest among the schizotypy (OR=2.3, 95% CI 2.0–2.6) subgroup, and was also increased in dysphoric pseudo-schizotypy (OR=1.6, 95% CI 1.5–1.8) and introverted pseudo-schizotypy (OR=1.4, 95% CI 1.3–1.5), relative to the no-risk group.

**Discussion:**

We provide evidence for distinct subtypes of children expressing different forms of schizotypy among a large Australian sample from the general population. The subgroup of children labeled ‘schizotypy’ (6%) characterized by high levels of cognitive disorganisation, impulsive non-conformity, introversion, and self-other disturbance may be at highest risk for developing schizophrenia or other mental illness in adulthood, and had a greater likelihood of childhood maltreatment and parental mental illness history, than other ‘pseudo-schizotypy’ groups.

**Reference:**

1. Carr, V.J., Harris, F., Raudino, A., Luo, L., Kariuki, M., Liu, E., Tzoumakis, S., Smith, M., Holbrook, A., Bore, M., Brinkman, S.A., Lenroot, R.K., Dix, K., Dean, K., Laurens, K.R., Green, M.J. (2016) Cohort Profile: The New South Wales Child Development Study (NSW-CDS) – An Australian multi-agency, multi-generational, longitudinal record linkage study. 6:e009023 doi:10.1136/bmjopen-2015–009023.

